# Disentangling body image: The relative associations of overvaluation, dissatisfaction, and preoccupation with psychological distress and eating disorder behaviors in male and female adolescents

**DOI:** 10.1002/eat.22592

**Published:** 2016-08-19

**Authors:** Deborah Mitchison, Phillipa Hay, Scott Griffiths, Stuart B. Murray, Caroline Bentley, Kassandra Gratwick‐Sarll, Carmel Harrison, Jonathan Mond

**Affiliations:** ^1^ Department of Psychology Macquarie University Sydney New South Wales Australia; ^2^ School of Medicine Western Sydney University Sydney New South Wales Australia; ^3^ Centre for Health Research, Sydney, Western Sydney University New South Wales Australia; ^4^ Department of Psychology University of Canberra Canberra Australian Capital Territory Australia; ^5^ Department of Psychiatry University of California San Francisco California; ^6^ Research School of Psychology Australian National University Canberra Australian Capital Territory Australia

**Keywords:** mediation, community‐based, body image, dissatisfaction, preoccupation, overvaluation, boys, girls, eating disorder behaviours

## Abstract

**Objective:**

The distinctiveness and relative clinical significance of overvaluation, dissatisfaction, and preoccupation with body weight/shape remains inconclusive. This study sought to add to the evidence by testing associations between these three body image constructs and indicators of clinical significance.

**Method:**

Male and female secondary students (*N* = 1,666) aged 12–18 years completed a survey that included measures of dissatisfaction with, overvaluation of, and preoccupation with weight/shape, psychological distress, eating disorder behaviors, and basic demographic information. Conditional process analysis was employed to test the independent and mediating effects of overvaluation, dissatisfaction, and preoccupation on distress, dietary restraint, and objective binge eating.

**Results:**

Overvaluation, dissatisfaction, and preoccupation were highly correlated (*r* = 0.47–0.84). In girls, preoccupation demonstrated the strongest independent and mediating effects on distress, dietary restraint, and binge eating; whereas neither the direct or indirect effects of dissatisfaction on distress and overvaluation on binge eating were significant. Among boys however, the direct and indirect effects of overvaluation, dissatisfaction, and preoccupation on distress and eating disorder behaviors were relatively equal.

**Discussion:**

Preoccupation with weight/shape may be particularly clinically significant in girls, whereas all constructs of body image disturbance may be equally clinically significant in boys. The findings are consistent with the view that these constructs, while closely related, are distinct. © 2016 Wiley Periodicals, Inc.(Int J Eat Disord 2017; 50:118–126)

## Introduction

Body image disturbance is core to eating disorders, and theorized to underpin maladaptive eating and weight‐loss behaviors.^1^ Constructs that are commonly referred to include dissatisfaction, overvaluation, and to a lesser extent, preoccupation with weight and/or shape.^2^ Overvaluation of weight and/or shape (overvaluation) is defined as the assignment of excessive importance to body weight and/or shape in evaluating one's self worth, whereas dissatisfaction with weight/shape (dissatisfaction) is defined as the negative evaluation of one's body weight and/or shape. Preoccupation is defined as excessive thinking about weight and/or shape.

Despite the prominence of body image disturbance in eating disorders (e.g., in theoretical and diagnostic models), its conceptualization remains ill‐defined, with distinct aspects/terms used interchangeably or referred to using “catch‐all” phrases such as “weight/shape concerns.”^3^, ^4^ For instance, in the Diagnostic and Statistical Manual for Mental Disorders (DSM‐5),^5^ overvaluation of weight or shape, referred to as the “undue influence of body weight or shape on self‐evaluation” (p. 339^5^), is included among the diagnostic criteria for anorexia nervosa but need not be present if there is poor insight into the seriousness of the low weight. On the other hand, overvaluation of weight or shape is a discrete and core criterion for the diagnosis of bulimia nervosa.^5^ There is currently no mention of body image disturbance in the diagnostic criteria for binge eating disorder, although this absence is the subject of debate.^6^, ^7^, ^8^ In strictly applying these criteria, body image disturbance is only really an essential criterion in bulimia nervosa; however, this does not reflect clinical reality, in which body image disturbance is widespread among these disorders. Body image disturbance is also ill‐defined when used as an exclusionary criterion. For instance, in the diagnosis of avoidant/restrictive food intake disorder (ARFID) the DSM‐5 stipulates that there must be no evidence of “…a disturbance in the way in which one's body weight or shape is experienced” (p. 334^5^), with no definition of what the experience of weight or shape entails.

In comparison to dissatisfaction, overvaluation has received relatively greater attention as an indicator of body image disturbance^9^ and is central to the transdiagnostic cognitive behavioral model of eating disorders.^1^ In line with this, it has been suggested that dissatisfaction is more “normative” than overvaluation. This however may be truer for women than for men. Evidence from epidemiological studies suggests that marked dissatisfaction (15% in men, 40% in women^10^, ^11^) is indeed more common than marked overvaluation (14% in men, 23% in women^12^) in women, although the constructs may be similarly prevalent among men. Regardless of prevalence, neither construct should be considered benign, with both overvaluation and dissatisfaction having been shown to be associated with significant impairment in quality of life,^10^, ^13^ and overvaluation also predictive of reduced productivity.^14^ These findings indicate the clinical significance of these aspects of body image disturbance. Of the few studies that have assessed gender differences, Griffiths et al.^11^ found that dissatisfaction was associated with higher levels of psychological distress and quality of life impairment in men compared to women, whereas Mitchison et al.^12^ found that overvaluation was associated with greater quality of life impairment in women compared to men. Thus further work is required to determine whether the clinical significance of these constructs varies according to gender.

There has been little empirical investigation of differences between dissatisfaction and overvaluation to disentangle their relative clinical significance. The DSM defines clinical significance as the association of a symptom or set of symptoms with either marked psychological distress or impairment in functioning. Other indicators of clinical significance, particularly of cognitive constructs, is the degree to which they are associated with maladaptive behaviors (in eating disorders, this may include extreme weight loss or gain behaviors or binge eating). Treatment studies with bulimia nervosa^9^ and gastric bypass^15^ patients have found that improvements in dissatisfaction are associated with different clinical outcomes compared to improvements in overvaluation. Specifically, improved levels of weight/shape dissatisfaction following treatment has been associated with improvements in both negative affect^9^, ^15^ and self‐esteem,^15^ whereas improvements in overvaluation has been associated with improved self‐esteem only.^9^, ^15^

Community‐based studies have also found differences. For instance, overvaluation has been associated with attempts to gain muscle in adolescents,^16^ whereas dissatisfaction has been associated with weight loss behavior and binge eating in pre‐early adolescents,^16^, ^17^ and higher levels of distress in women.^18^ A general population study of women reported that the additive effects of overvaluation and dissatisfaction were greater than the effect of either alone in terms of association with psychological distress.^18^ Finally, a longitudinal study with pre‐adolescents found that weight/shape concerns were more predictive of overvaluation over time, than vice versa, which demonstrates a possible developmental trajectory of body image disturbance, with weight/shape concerns providing a platform for the later development of overvaluation.^17^ The two early‐adolescent studies included a relatively even number of boys and girls, and both found that gender did not moderate associations between dissatisfaction or overvaluation with eating disorder behaviors or psychosocial variables.^16^, ^17^

Limitations of these studies include multidimensional measures of body image disturbance such as “weight/shape concerns” that do not allow for complete isolation of specific constructs,^15^, ^17^ the use of discrepancy‐based measures of dissatisfaction, which subtract ideal weight from current weight without controlling for distress,^16^, ^17^ and the lack of mediation analysis to tease out how body image disturbance constructs may interact in their association with eating disorder behavior, distress, and functional impairment.

Very few studies have examined the clinical significance of preoccupation. This is despite preoccupation being a core feature in widely used eating disorder assessment measures (e.g., the Eating Disorder Examination^2^ and the Eating Attitudes Test^19^), and more severe in people with eating disorders than with body dysmorphic disorder^20^ (a disorder in which preoccupation is a diagnostic criterion). Community‐based studies of women have found that excessive preoccupation with appearance contributes to variance in eating disorder pathology,^21^, ^22^ and distress and impairment in quality of life beyond that attributed by other eating disorder symptoms.^21^ In the only study to date to compare the role of preoccupation with other body image constructs, Wade et al. reported that overvaluation and preoccupation shared substantial genetic risk, whereas dissatisfaction was genetically more closely related to body mass index.^2^ Given that known eating disorder risk factors include both sociodemographic and genetic variables,^23^ the role of epigenetic processes (the interaction of these variables) in the development of body image disturbance should also be explored.

In sum, existing evidence bearing on the association between overvaluation, dissatisfaction, and preoccupation is inconclusive and further research is required to elucidate the distinctiveness and clinical significance of these constructs. The goal of the current study was to add to this evidence by clarifying the independent and mediating associations between overvaluation, dissatisfaction, preoccupation, and three measures of clinical significance, namely, general psychological distress and two eating disorder behaviors, objective binge eating, and dietary restraint. As the evidence has been limited and inconclusive regarding the relationships between body image constructs, no a priori hypotheses were made.

## Method

### Sampling Procedures and Participants

Participants were recruited as part of the ACT Schools Mental Health Literacy Survey, details of which have been reported previously.^24^, ^25^, ^26^ Students attending 12 secondary schools in the Australian Capital Territory (ACT) were recruited to complete a survey of eating disorder symptoms and eating disorders “mental health literacy.” Participants all provided informed consent. Parents/guardians were also provided information about the study and the opportunity to exclude their child if they wished. The study was approved by the Human Research Ethics Committee at the Australian National University, the ACT Department of Education and Training, and the Catholic Education Office.

Students who attended class on the day of testing were invited to complete a questionnaire, in their classrooms, under the supervision of a teacher and one or more members of the research team. The questionnaire included measures of eating disorder symptoms, general psychological distress, and demographic information (age, first language, country of birth, residential postcode). Body mass index (BMI, kg m^2^) was derived from self‐reported height and weight.

Completed questionnaires were received from *N* = 1749 students (78.7% participation rate). Data for *n* = 9 participants who were younger than 12 years or older than 18 years of age, *n* = 70 participants with high levels of missing or corrupt data, and *n* = 4 participants who did not indicate their gender were excluded. The final sample therefore comprised *N* = 1666 12–18 year‐old boys (*n* = 531; 32%) and girls (*n* = 1135; 68.0%).^24^, ^25^, ^26^

### Measures

#### Body Image Disturbance

Dissatisfaction, overvaluation, and preoccupation were measured using five items of the Eating Disorder Examination Questionnaire (EDE‐Q),^27^ a self‐report measure of cognitive and behavioral eating disorder features experienced over the past 28 days. The EDE‐Q has been validated in community and clinical samples.^28^ Norms for Australian adolescents have been reported previously,^25^, ^29^ together with evidence of adequate scale score reliability.^30^ For the assessment of dissatisfaction and overvaluation, the average score of two items addressing these constructs (dissatisfaction with weight, dissatisfaction with shape; overvaluation of weight, overvaluation of shape) was used, whereas a single item was used to assess preoccupation. The wording of the questions was as follows: “How dissatisfied have you been with your weight [shape]?” (dissatisfaction), “Has your weight [shape] influenced how you think about (judge) yourself as a person?” (overvaluation), and “Has thinking about shape or weight made it very difficult to concentrate on things you are interested in (for example, working, or following a conversation)?” (preoccupation).

Responses to these questions are given on a 7‐point (0 to 6), Likert‐type scale, with higher scores indicating greater severity of body image disturbance. For dissatisfaction and overvaluation, 0 = “not at all” and 6 = “markedly.” For preoccupation, 0 = “no days” and 6 = “every day.” These singular items have been used previously in population and community‐based research to indicate the presence of different facets of body image disturbance.^10^, ^12^, ^14^, ^18^

#### General Psychological Distress

The Kessler Psychological Distress Scale (K‐10)^31^, ^32^ was used to measure the frequency of anxiety and/or depressive symptoms during the past 4 weeks. Respondents are asked to answer 10 questions on a five‐point Likert‐type scale, indicating the presence of symptoms from “none of the time” (1) to “all of the time” (5). Scores range from 10 to 50, with higher scores indicating higher levels of distress, and scores of 30 or higher being indicative of clinical levels of distress. The K‐10 has demonstrated high internal consistency and validity in predicting clinically significant levels of distress in general population samples.^26^, ^31^, ^32^ Cronbach's alphas for boys and girls in the present study sample were 0.87 and 0.91, respectively.

#### Objective Binge Eating

One of the behavioral items on the EDE‐Q, described above, was used to assess the number of days in the past 28 days participants had engaged in objective binge eating. Participants are asked to indicate the number of days that they had eaten an unusually large amount of food and at the same time experienced a sense of loss of control whilst eating. The item has been used previously in population and community‐based research (e.g., Refs. 12 and 14) and was chosen as its measurement does not crossover with the items on the EDE‐Q used to measure the body image constructs above.

#### Dietary Restraint

The restraint subscale of the EDE‐Q, described above, was used to measure the severity of dietary restraint. The subscale is calculated by averaging the scores on five items (range: 0 to 6, with higher scores indicative of greater levels of restraint). These items ask participants to indicate the extent to which they have tried to limit their eating, fasted, followed diet rules, and desired an empty stomach over the past 28 days. Again, this subscale was chosen as a measure of eating disorder behavior, with its items being independent of the items on the EDE‐Q used to assess body image constructs in this study. In addition to objective binge eating and dietary restraint, it would also have been useful to assess purging behavior. However as this is a community‐based study, the prevalence of this behavior was too low to permit meaningful analysis.

### Data Analysis

#### Preliminary Analyses

*χ* ^2^, ordinal logistic regression, and analysis of variance were employed to compare boys and girls with respect to the main variables of interest (levels of distress, objective binge eating frequency, dietary restraint, overvaluation, dissatisfaction, preoccupation) and other demographic characteristics (BMI, age, first language, and country of birth). Spearman correlation coefficients were computed to determine the associations between each of the body image constructs (overvaluation, dissatisfaction, preoccupation) and outcome variables (psychological distress, objective binge eating frequency, dietary restraint). Partial correlation coefficients were computed for each body image construct with each outcome variable, controlling for the remaining body image constructs (available in the Supporting Information file). Data were stratified by gender in all of these analyses.

#### Imputation of Missing Data

A moderate number of cases (17.1%) had incomplete data on the variables used in the analysis of the present study, although only 2.0% of total data values were missing (*n* = 32 for the K‐10, *n* = 0 for objective binge eating, *n* = 0 for dietary restraint, *n* = 10 for dissatisfaction, *n* = 5 for preoccupation, *n* = 3 overvaluation, and *n* = 4 for gender). The automatic multiple imputation method provided for in SPSS was used, with linear regression employed for scale variables. The following were entered as both dependent and predictor variables: K‐10 total score, dissatisfaction, preoccupation, overvaluation, gender, postcode, age, first language, country of birth, and BMI category. Five imputations were computed, and the pooled results are reported.

#### Conditional Process Analysis

Preliminary analysis established that the residuals of the linear regression including all variables in the model were normally distributed. Conditional process analysis,^32^, ^33^ a cross‐sectional mediation method, was conducted separately for boys and girls to examine the direct and indirect effects of overvaluation, dissatisfaction, and preoccupation on psychological distress, objective binge eating, and dietary restraint. Demographic characteristics (age, first language, country of birth, postcode) and BMI were included as covariates in these analyses. Analyses were conducted using SPSS version 23.0. Results were considered significant at *p* < 0.05.

## Results

Descriptive statistics are shown in **Table** 1. As can be seen, compared to the boys, the girls in this study were older, more likely to be a healthy weight, had a higher binge eating frequency, and higher scores on the K10, Dietary Restraint subscale of the EDE‐Q, and the variables of dissatisfaction, overvaluation, and preoccupation.

**Table 1:** Characteristics of male and female participants

	Males (*n* = 531)	Females (*n* = 1135)		
	*M* (SD)	*M* (SD)	*F* (*df*), *p*	Partial η^2^
K‐10	18.54 (8.49)	22.65 (8.49)	94.25 (1, 1632), < 0.001	0.055
OBE (days/past month)	0.70 (2.95)	1.99 (4.89)	31.65 (1, 1664), < 0.001	0.019
Dietary restraint	0.54 (0.97)	1.48 (1.57)	159.43 (1, 1664), < 0.001	0.087
Dissatisfaction	0.91 (1.38)	2.62 (2.08)	295.80 (1, 1648), < 0.001	0.152
Overvaluation	0.93 (1.30)	2.41 (2.02)	234.17 (1, 1648), < 0.001	0.124
Preoccupation	0.30 (0.92)	1.21 (1.82)	118.48 (1, 1648), < 0.001	0.067
Age	14.84 (1.70)	15.51 (1.63)	59.02 (1, 1662), < 0.001	0.034
	***n* (%)**	***n* (%)**	**χ^2^ (*df*), *p***	**OR (95% CI)**
English first language	475 (89.6)	1024 (90.7)	0.48 (1), 0.488	—
Born in Australia	457 (86.7)	998 (89.0)	1.85 (1), 0.174	—
Weight status			8.70 (3), 0.034	
Underweight	36 (7.5)	77 (7.6)	—	1.91 (0.98, 3.73)
Healthy Weight	369 (76.7)	821 (82.5)	—	1.99 (1.14, 3.45)
Overweight	51 (10.6)	81 (8.0)	—	1.42 (0.75, 2.70)
Obese	25 (5.2)	28 (2.8)	—	—

NB: OBE = objective binge eating. K‐10 = Kessler Psychological Distress Scale. Dietary Restraint measured using the Eating Disorder Examination Questionnaire Restraint subscale. Missing data were observed for the K‐10 (*n =* 32 cases), Dissatisfaction/Overvaluation/Preoccupation (*n* = 16 cases), age (*n =* 2), BMI (*n* = 182 cases), English First Language (*n* = 11 cases), and Born in Australia (*n* = 22 cases) variables.

### Correlational Analysis

As can be seen in **Table** 2, correlations between body image disturbance constructs and psychological distress (*r* = 0.47 to 0.51), dietary restraint (*r* = 0.40 to 0.70), and objective binge eating (*r* = 0.25 to 0.38) were significant, moderate to large, and similar in size. Further, overvaluation, dissatisfaction, and preoccupation were all significantly correlated (*r* = 0.47 to 0.84).

**Table 2:** Spearman correlations between body image disturbance constructs, distress, and eating disorder behaviors for male and female adolescents

	Dissatisfaction	Overvaluation	Preoccupation	Distress	OBE	Dietary Restraint
Dissatisfaction	—	0.67***	0.47***	0.35***	0.32***	0.40***
Overvaluation	0.84***	—	0.47***	0.37***	0.30***	0.41***
Preoccupation	0.66***	0.66***	—	0.28***	0.25***	0.44***
Distress	0.47***	0.51***	0.49***	—	0.26***	0.22***
OBE	0.38***	0.37***	0.35***	0.28***	—	0.27***
Dietary Restraint	0.70***	0.69***	0.66***	0.42***	0.34***	—

NB: Correlation coefficients for males are shaded. ^***^ *p* < 0.001, ^**^ *p* < 0.01.

### Conditional Process Analysis—Direct and Indirect Effects of Body Image Disturbance on Clinical Significance Outcomes

#### Psychological Distress

The direct effects displayed in **Table** 3 indicate the degree of independent association between each of the body image constructs (overvaluation, dissatisfaction, preoccupation) and each outcome variable (psychological distress, dietary restraint, objective binge eating), whilst controlling for concomitant effects of the other body image constructs.

**Table 3:** Direct effects from the conditional process analysis of overvaluation, dissatisfaction, and preoccupation on distress, dietary restraint, and binge eating among male (a) and female (b) adolescents

a. Males									
	Psychological Distress			Dietary Restraint			Objective Binge Eating		
**Predictor**	*Β*	S.E.	90% C.I.	*Β*	S.E.	90% C.I.	*Β*	S.E.	90% C.I.
Overvaluation	1.12***	0.29	0.65, 1.60	0.15***	0.04	0.09, 0.21	0.43**	0.13	0.21, 0.65
Dissatisfaction	0.75**	0.29	0.27, 1.23	0.09*	0.04	0.03, 0.15	0.11	0.14	−0.12, 0.33
Preoccupation	0.89**	0.36	0.30, 1.48	0.40***	0.04	0.33, 0.48	0.31†	0.17	0.03, 0.58
	*R* ^2^ *=* 0.20			*R* ^2^ *=* 0.43			*R* ^2^ *=* 0.09		
	*F* (8, 522) = 16.18, *p* < 0.001			*F* (8, 522) = 48.36, *p* < 0.001			*F* (8, 522) = 6.70, *p* < 0.001		

b. Females									
	Psychological Distress			Dietary Restraint			Objective Binge Eating		
**Predictor**	*Β*	S.E.	90% C.I.	*Β*	S.E.	90% C.I.	*Β*	S.E.	90% C.I.
Overvaluation	1.10***	0.2	0.77, 1.43	0.20***	0.03	0.15, 0.24	0.13	0.13	−0.08, 0.35
Dissatisfaction	0.13	0.2	−0.20, 0.45	0.14***	0.03	0.10, 0.19	0.40**	0.13	0.19, 0.61
Preoccupation	1.41***	0.16	1.15, 1.67	0.36***	0.02	0.32, 0.40	0.55***	0.10	0.38, 0.71
	*R* ^2^ *=* 0.20			*R* ^2^ *=* 0.59			*R* ^2^ *=* 0.14		
	*F* (8, 1126) = 16.18, *p* < 0.001			*F* (8, 1126) = 205.12, *p* < 0.001			*F* (8, 1126) = 23.31, *p* < 0.001		

^***^ *p* < 0.001, ^**^ *p* < 0.01, ^*^ *p* < 0.05, ^†^ *p* < 0.10.

Among girls, preoccupation and overvaluation, but not dissatisfaction, had a significant direct effect on psychological distress. When the partial correlations were compared (available in the Supporting Information file), preoccupation was found to have a significantly larger correlation with distress compared to overvaluation, and overvaluation in turn had a larger correlation with distress compared to dissatisfaction. Inspecting the indirect effects, preoccupation (*β* = 0.54) and overvaluation (*β* = 0.25) were each found to significantly mediate the relationship between each other and distress, as well as the relationship between dissatisfaction and distress (*β* = 0.38 and 0.76, respectively).

Among boys, dissatisfaction, overvaluation, and preoccupation each had a significant direct effect on psychological distress, and the partial correlations with distress did not significantly differ between any of the constructs. Overvaluation (*β* = 0.19–0.64), dissatisfaction (*β* = 0.37–0.41), and preoccupation (*β* = 0.28) all mediated the effects of each other on psychological distress, with the exception that preoccupation was not found to mediate the association between overvaluation and distress.

#### Objective Binge Eating

Among girls, preoccupation and dissatisfaction, but not overvaluation, were found to have a significant direct effect on the frequency of objective binge eating in the past 28 days. The partial correlation between preoccupation and objective binge eating was significantly larger than the correlation between dissatisfaction and objective binge eating, which in turn was larger than the correlation between overvaluation and objective binge eating. The indirect effects indicated that preoccupation and dissatisfaction mediated the effects of each other (*β* = 0.15 and 0.07, respectively) and overvaluation (*β* = 0.21 and 0.29, respectively) on objective binge eating frequency.

Among boys, preoccupation and overvaluation, but not dissatisfaction, had significant direct effects on objective binge eating, although the size of the partial correlations between each of these constructs and binge eating did not differ significantly. Overvaluation mediated the effects of both dissatisfaction (*β* = 0.25) and preoccupation (*β* = 0.07) on binge eating.

#### Dietary Restraint

Among girls, all three body image constructs had a significant direct effect on dietary restraint; however the partial correlation was significantly stronger between preoccupation and dietary restraint, compared to the correlations between both overvaluation and dissatisfaction and dietary restraint. Preoccupation (*β* = 0.10–0.14), overvaluation (*β* = 0.05–0.14), and dissatisfaction (*β* = 0.02–0.10) each mediated the effects of each other on dietary restraint.

A similar pattern was observed among boys, whereby all direct effects on dietary restraint were significant; preoccupation emerged as having the strongest independent association with dietary restraint; and preoccupation (*β* = 0.04–0.14), overvaluation (*β* = 0.03–0.09), and dissatisfaction (*β* = 0.04–0.05) each mediated the effects of each other on dietary restraint.

## Discussion

This study sought to disentangle the relative independent and mediating relationships between various body image constructs and indicators of clinical significance, in a population‐based sample of adolescents. We were also interested to consider similarities and differences in these effects across gender. We found that preoccupation with weight and shape emerged as a particularly potent predictor of distress and eating disorder behaviors in girls and dietary restraint in boys, whereas the preoccupation, overvaluation, and dissatisfaction with weight and shape were relatively equal contributors to distress and binge eating in boys.

The current findings are consistent with the view that under the umbrella of ‘body image disturbance’, dissatisfaction, overvaluation, and preoccupation are distinct constructs, particularly for young girls. In addition, the findings indicate the need for caution in assuming that either of these constructs has the same meaning, in terms of their clinical significance, in boys as in girls.^34^, ^35^ To classify a mental disorder, the DSM stipulates that the symptom/s must result in significant psychological distress (or functional impairment). In female adolescents, it would appear that dissatisfaction with one's weight or shape is not sufficient to cause distress, although as other research suggests, it may well be necessary.^18^ In boys, by contrast, dissatisfaction may be associated with distress regardless of whether overvaluation or preoccupation is also present. Evidence suggests that young men may hold more stigmatizing views of body image disorders than young women, perceiving thinness‐oriented concerns and behaviors as being less masculine.^36^ It is possible, therefore, that boys in the current study who were aware of their weight/shape dissatisfaction may have been more prone to self‐criticism and, in turn, higher levels of distress. Alternatively, the observed relationship in this study between preoccupation, overvaluation, and dissatisfaction (in boys) and scores on the K‐10 may be indicative of comorbid symptoms of anxiety or depression, which may increase vulnerability to the development of eating disorder symptoms.^37^ Still others have suggested a cyclical exacerbating relationship between distress and body image/eating disorder pathology.^38^

In regard to eating disorder behaviors, the picture is somewhat different. For both boys and girls, preoccupation exacted a powerful independent effect whilst also mediating the effects of overvaluation and dissatisfaction on dietary restraint. On the other hand, preoccupation retained the strongest direct and indirect effects on binge eating in girls, whereas overvaluation had the strongest effects on binge eating in boys. Indeed, among girls, no direct or indirect effects of overvaluation on binge eating were observed. Thus an additional implication of the current research is that further consideration needs to be given to the status of preoccupation as a component of body image disturbance. Interestingly, the current study found that the direct and indirect effects of preoccupation on psychological distress and eating disorder behaviors were often stronger than that observed for overvaluation. These findings may indicate that young people may compulsively engage in eating disorder behaviors as a response to (obsessive) preoccupation, similar to patterns observed in obsessive compulsive disorder. In contrast to dissatisfaction and overvaluation, preoccupation has rarely been considered in research addressing the role of body image disturbance in the onset and maintenance of eating disorder pathology. Given the role of preoccupation in other disorders assumed to be closely related to eating disorders, such as body dysmorphic disorder and obsessive compulsive disorder,^5^ future research investigating the role of preoccupation in eating disorders is warranted.

Because the current research was conducted in a general population sample, there are no direct implications for diagnostic formulation. Nevertheless, given the associations found with distress and behavioral eating disorder symptoms, the current findings suggest that in addition to overvaluation,^1^ preoccupation may also be particularly central to eating disorder maintenance. In light of the relatively equal status of dissatisfaction, overvaluation, and preoccupation among boys in this study, future work may consider sex‐specific revisions of theoretical models of eating disorder maintenance, such as the transdiagnostic model,^1^ in which overvaluation alone is posited as central. This would be timely given the current climate of increasing interest in the phenomenology of body image disturbance and eating pathology in males (e.g., muscle dysmorphic‐type symptoms) and in the applicability of current diagnostic criteria and assessment instruments for eating disorders to males more generally.^34^, ^35^

Some, tentative implications of the current findings for clinical practice might also be mentioned. Given the findings related to preoccupation, interventions that help clients to redirect attention away from thoughts about weight and shape (such as attention and mindfulness based training) might be developed and prioritized in treatment. For young male clients, on the other hand, dissatisfaction, overvaluation, and preoccupation may be equally important targets for intervention. At the same time, it needs to be remembered that these different manifestations of body image disturbance, as currently conceptualized, are highly correlated and may be more likely than not to manifest at the same time.^18^

### Strengths and Limitations

Strengths of this study include the recruitment of a large, community‐based sample of boys and girls and the use of a novel method, namely, conditional process analysis,^33^ to elucidate the associations of dissatisfaction, overvaluation, and preoccupation with indicators of clinical significance. To our knowledge, this is the first study to attempt to disentangle components of body image disturbance using this methodology.

This study employed recently developed sophisticated methods by Hayes for conducting mediation analysis in cross‐sectional research.^33^, ^39^ This is a method around which there is considerable debate, and we acknowledge that the primary limitation of the current research is its cross‐sectional study design, the use of which precludes any firm conclusions as to the direction of the observed associations. A longitudinal replication will be an important future direction in order to verify the current findings. Another limitation was the reliance on just one or two items to assess the respective body image constructs. Measuring whole constructs with few items potentially reduces construct validity and reliability. On the other hand, the fact that significant differences emerged in analyses between these constructs without large confidence intervals indicates that participants were distinguishing between the body image items in a consistent manner. More generally, research seeking to disentangle key components of body image disturbance is hampered by a lack of reliable and valid measures that adequately isolate these constructs. An important direction for future research will be the development of standardized measures of both preoccupation and overvaluation of weight and shape, given their theoretical prominence and clinical significance as supported by the current study's findings.

Additional limitations include the use of a measure of body image disturbance, namely, the EDE‐Q, that was developed with thinness‐oriented psychopathology in mind and which may therefore be relatively insensitive to weight/shape concerns as these more commonly present in boys (e.g., distress associated with being too small)^34^, ^35^; and self‐report of height and weight, which is less valid than anthropometric measurements.

Although it would have been possible to identify subgroups of participants with a probable eating disorder diagnosis, the relatively small number of boys precluded analysis of this kind. Ideally, both community and clinical samples would be used in future research to assess the applicability of the current findings to the development of clinical eating disorders. In research employing clinical samples, it will be important to include both eating disorders in which body image disturbance is deemed a core feature (e.g., anorexia nervosa, bulimia nervosa) and eating disorders in which it is not (e.g., binge eating disorder, avoidant/restrictive food intake disorder), as well as other disorders involving body image disturbance (e.g., body dysmorphic disorder, muscle dysmorphia). It will also be important to consider other indices of clinical significance aside from psychological distress, such as impairment in role (e.g., social, occupational) functioning, in future research. Finally, it would be of interest to replicate the current findings in an adult sample. It is quite possible that a different pattern of findings would be observed in adult vs. adolescent populations, as has been observed in research addressing the status of overvaluation among individuals with a probable diagnosis of binge eating disorder.^40^

## Supporting information

Supporting Information
