# Supplementary material for: Disentangling body image: The relative associations of overvaluation, dissatisfaction, and preoccupation with psychological distress and eating disorder behaviors in male and female adolescents
Source: Int J Eat Disord. 2016 Aug 19;50(2):118–26. doi: 10.1002/eat.22592 (PMC6585604; doi:10.1002/eat.22592)
Supplement: Supplementary file 1 — Supporting Information [file EAT-50-118-s001.doc]

|  | **Dissatisfaction (D)** | **Overvaluation (O)** | **Preoccupation (P)** | **Significance Testing** |
| --- | --- | --- | --- | --- |
| **FEMALES** |  |  |  |  |
| Distress | 0.03 | 0.17*** | 0.25*** | P > O > D |
| OBE | 0.09* | 0.02 | 0.16*** | P > D > O |
| Dietary Restraint | 0.15*** | 0.21*** | 0.43*** | P > D = O |
| **MALES** |  |  |  |  |
| Distress | 0.10** | 0.17*** | 0.11* | D = O = P |
| OBE | 0.03 | 0.14** | 0.10* | D = O = P |
| Dietary Restraint | 0.12** | 0.20*** | 0.37*** | P > D = O |
| OBE = objective binge eating | | | | |

Supplemental Table. *Partial Correlations between Body Image Disturbance Constructs and Outcome Variables for Male and Female Adolescents*
